# Mucinous cystadenoma of the pancreas with predominant stroma creating a solid tumor

**DOI:** 10.1186/1477-7819-3-59

**Published:** 2005-09-07

**Authors:** Won Ae Lee

**Affiliations:** 1 Department of Pathology, College of Medicine Dankook University, Cheonan, Republic of Korea

## Abstract

**Background:**

Mucinous cystic neoplasm (MCN) of the pancreas is basically cystic epithelial neoplasm, unilocular or multilocular, occurring almost exclusively in women.

**Case presentation:**

A 51-year-old female presented with a pancreatic mass incidentally found on the abdominal computed tomography. She underwent distal pancreatectomy. The sectioned surface of the pancreas revealed a circumscribed, whitish gray ovoid firm mass with some cystic spaces. Microscopically, glandular or small cystic structures were scattered in the predominant stroma creating a solid appearance. The subepithelial stromal component was composed of cytologically bland looking spindle cells, which resembled ovarian stroma. The stromal cells were reactive to CD 34, vimentin, progesterone receptor and calretinin. The microscopy was consistent with mucinous cystadenoma of the pancreas.

**Conclusion:**

This case of mucinous cystadenoma of the pancreas showed very interesting pathology: It was solid rather than cystic, and accompanied by abundant benign transitional epithelia, which was a very unusual and novel finding in the mucinous cystic neoplasm of the pancreas.

## Background

Mucinous cystic neoplasm (MCN) of the pancreas is a cystic neoplasm, unilocular or multilocular, occurring almost exclusively in women. The overwhelming majority of cases occur in the body-tail of the pancreas. MCNs show two distinct components: an inner mucinous epithelial layer and an outer dense cellular ovarian-type stromal layer [1-5].

The case reported here is an exceptional case of MCN of the pancreas with predominant stromal component creating a solid tumor, and abundant transitional cell differentiation.

## Case presentation

**Figure 1:**
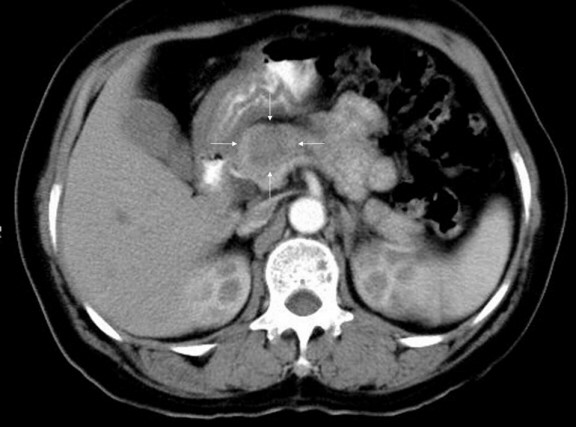
Contrast enhanced abdominal computed tomography showing an ill defined mass revealing faint inhomogeneous low density in the body of the pancreas.

A 51-year-old female presented with a pancreatic mass found incidentally on the abdominal computed tomography for routine health examination. The mass was located in the body of the pancreas and was ill-defined with faint inhomogeneous low density at both the arterial and the venous phases of computerized tomography (CT) scan (Figure 1). Endoscopic retrograde cholangiopancreaticography showed mild indentation and slight irregularity of the neck portion of the main pancreatic duct suggesting extrinsic compression of the main pancreatic duct. The patient underwent distal pancreatectomy.

**Figure 2:**
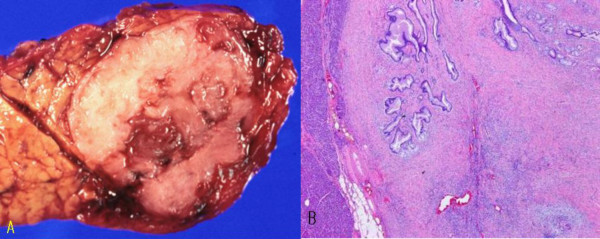
**A. Macroscopic photograph of the tumor**. Sectioned surface reveals a non-encapsulated, circumscribed, gray-white, firm, solid mass with partially lobulate margin and irregular small cysts. **B. Low power view of microscopic features of the tumor**. Cystic or glandular epithelial components are set in abundant dense stroma (hematoxylin and eosin, × 40)

**Figure 3:**
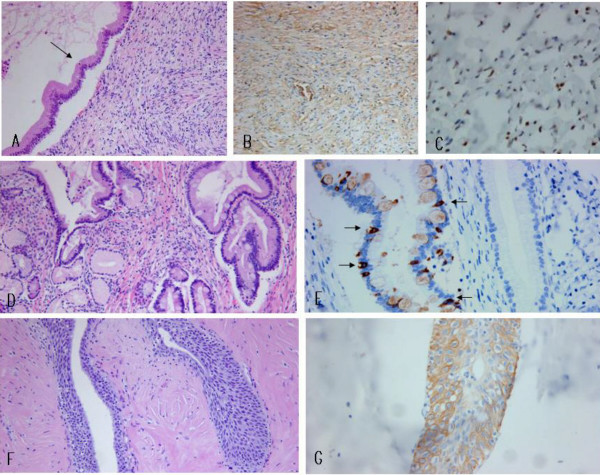
**Microscopic photograph of the tumor**. A. Cystic structure is lined by benign columnar mucinous epithelial cells (arrow). Subepithelial stroma is composed of bland looking spindle cells (asterisk) mimicking ovarian stroma (hematoxylin and eosin, × 200). B. Stromal cells are immunoreactive against calretinin (× 200). C. Stromal cells are reactive against progesterone receptor (× 400). D. Columnar epithelia are accompanied by intestinal metaplasia with scattered goblet cells (right) as well as pseudopyloric metaplasia (left) (hematoxylin and eosin, × 200). E. Scattered endocrine cells (arrows) are highlighted by reactivity to chromogranin A (× 400). F. Cysts are lined by benign transitional-type epithelium, beneath which the stroma is hyalinized (hematoxylin and eosin, × 200). G. Transitional cells are immunoreactive to high molecular weight cytokeratin (× 400).

The sectioned surface of the resected pancreas revealed a non-encapsulated, partially lobulate, whitish gray ovoid firm mass with 2.5 cm in its greatest dimension. The mass was predominantly solid with some cystic spaces, which contained mucinous fluid (Figure 2A). The mass was not communicated with pancreatic ductal system. Microscopically, the tumor was circumscribed with focal entrapped normal pancreatic acini in the periphery of the tumor. Glandular or small cystic structures were scattered in the predominant stroma, which created the tumor's solid appearance (Figure 2B). The glands or cysts were lined by a single layer of tall columnar epithelial cells, which revealed basally located nuclei and abundant intracellular mucin that was positively stained on periodic acid Schiff and alcian blue. The subepithelial stroma was cellular and composed of bland looking spindle cells mimicking ovarian stroma (Figure 3A). The columnar epithelia contained occasional goblet cells and endocrine cells and revealed abundant pseudopyloric metaplasia (Figure 3D). Some cysts were lined by bland looking polygonal or ovoid stratified cells suggestive of benign transitional epithelia (Figure 3F). The stroma adjacent to transitional epithelia was hypocellular and densely hyalinized compared to that adjacent to the columnar epithelia. As a whole the stroma was composed of cytologically bland looking spindle cells, which had variable cellularity, areas of extensive stromal hyalinization, no cytologic atypia and no mitoses.

Immunohistochemical studies were performed on formalin-fixed, paraffin-embedded tissue sections by the avidin-biotin peroxidase complex method. The primary antibodies used were as follow as; Pancytokeratin (1:50, AE and AE3, Zymed, San Francisco, USA), high molecular weight cytokeratin (1:100, 34betaE12, DAKO, Denmark), cytokeratin 7 (1:50, OV-TL 12/30, DAKO), cytokeratin 20 (1:40, Ks20.8, DAKO), calretinin (1:50, polyclonal, DAKO), estrogen receptor (1:40, 6F11, Novocastra, Newcastle, UK), progesterone receptor (1:80, 1A6, Novocastra), chromogranin A (1:200, LK2H10 and PHE5, NeoMarker, Fremint, CA, USA), CD34 (1:40, QBEnd 10, DAKO), vimentin (1:50, V-9, Biogenex, san Ramon, CA), α-smooth muscle actin (1:50, α-sm-1, Novocastra, Newcastle, UK), desmin (1:100, D33, DAKO), S-100 protein (1:150, B32.1, Biomeda, Foster city, CA), CD99 (1:100, HO36-1.1, NeoMarker, Fremint, CA), CD99 (1:100, HO36-1.1, Neomarker), c-kit (1:300, 104D2, DAKO). Both mucinous and transitional epithelia were diffusely immunoreactive to pancytokeratin and cytokeratin 7, but not reactive to cytokeratin 20 except for goblet cells. Transitional epithelia were diffusely reactive to high molecular weight cytokeratin (Figure 3G), whereas mucinous epithelia were not reactive to it. Scattered endocrine cells were highlighted by positive reactivity to chromogranin A (Figure 3E). The stromal cells were diffusely positive for CD 34 and vimentin, and focally positive for calretinin (Figure 3B) and progesterone receptor (Figure 3C), while negative for estrogen receptor, alpha-smooth muscle actin, desmin, c-kit, CD99, S100 protein and cytokeratin.

## Discussion

MCNs of the pancreas are defined by cystic epithelial neoplasms composed of columnar mucin-producing epithelium. This tumor occurs almost exclusively in women and shows no communication with the pancreatic duct system. According to the grade of dysplasia, tumors may be classified as adenoma, borderline and non-invasive or invasive carcinoma [1-5].

The present tumor was composed of mucin producing epithelium with an ovarian type stroma. The stroma was predominant overgrowing epithelial element and creating a solid tumor. From this point of view, the main differential diagnoses of the present case include MCN with sarcomatous stroma, benign mesenchymal tumors and solid-pseudopapillary neoplasm. MCN with sarcomatous stroma is a rare variant of MCNs associated with a malignant sarcomatous stroma. This sarcomatous stroma is extremely hypercellular, contains mitotic figures, and shows marked atypia and pleomorphism of stromal cells [6]. Unlike the sarcomatous stroma, the stroma of the present case showed neither significant cellular atypia nor mitoses. Primary mesenchymal tumors of the pancreas are extraordinarily rare. Examples of benign pancreatic mesenchymal tumors composed of bland looking spindle cells include inflammatory myofibroblastic tumor [7], extragastrointestinal stromal tumor [8] and solitary fibrous tumor [5]. The possibility of primary mesenchymal tumors of the pancreas can be excluded by reason of the following points. Although the predominant stromal component overgrew the epithelial element in the present case, the epithelial element was distributed in the periphery as well as the center of the tumor, suggesting that the epithelial component is a true tumor element, not non-neoplastic tissue entrapped in the tumor. Moreover, inflammatory myofibroblastic tumor can be excluded from the viewpoint of no significant mixture of chronic inflammatory cells in the present case. Recently, the pancreatic counterpart of gastrointestinal stromal tumor was described and was based on its c-kit positivity [8]. Although in the present case the spindle cells within stroma were reactive to CD34, they were not reactive to c-kit. To the best of my knowledge, only a case of solitary fibrous tumor of the pancreas has been reported in English literature [5]. Although the CD34 positivity of stromal cells in the present case mimicked solitary fibrous tumor, the characteristic histology of solitary fibrous tumor, which correspond to patternless growth of short fascicles, a short storiform arrangement of the spindle or ovoid cells and vascularization with slit-like space was not observed in the present case. In my investigation of CD34 reactivity for normal ovarian tissue, the normal ovarian stroma was also documented to be reactive to CD34 (unpublished data). Solid- pseudopapillary neoplasm is somewhat similar to the present case from the viewpoint of mixed solid and cystic features but different by reason that solid-pseudopapillary neoplasm is composed of monomorphic polyhedral cells forming solid and pseudopapillary structures [9].

The epithelial component of MCN is composed of columnar cells which can also reveal pseudopyloric, gastric foveolar, small and large intestinal, and squamous differentiation, as is also observed in ovarian MCN [1-4]. In the present case, abundant pseudopyloric and intestinal metaplasia as well as transitional differentiation was observed. Transitional epithelia were distinguished by high molecular weight cytokeratin positivity. The mixed mucinous cystadenoma and benign Brenner tumor were described in the ovary [10-12]. However, the accompaniment of transitional epithelium was not reported in MCN of the pancreas. The stroma adjacent to the transitional epithelium was denser and more hyalinized than that adjacent to the columnar epithelium. These histologic findings suggest the possibility of a pancreatic counterpart of mixed mucinous cystadenoma and benign Brenner tumor of the ovary.

The stromal component of MCN is composed of ovarian-type stroma which express vimentin and in a high proportion, progesterone receptor, estrogen receptor, calretinin, and alpha inhibin [11,13-15]. Calretinin has been shown to recognize testicular Leydig cells and hilar ovarian cells. In the present case, stromal cells were reactive for progesterone and calretinin. The possible derivation of the stromal component of MCNs from the ovarian primordium is supported by morphology, tendency to undergo luteinization, presence of hilar-like cells, and immunophenotypic sex cord-stromal differentiation [11,13-15]. It has been hypothesized that ectopic ovarian stroma incorporated during embryogenesis in the pancreas may release hormones and growth factors causing nearby epithelium to proliferate and form cystic tumors [11,13-15].

## Conclusion

This case is a pancreatic tumor showing very interesting and unusual pathology. According to the current WHO classification of pancreas, this case belongs to the mucinous cystadenoma. But this case was solid rather than cystic, and showed abundant benign transitional epithelia, which are very unusual and a novel finding that has not been described in MCNs of the pancreas. This histopathology is very similar to the mixed mucinous cystadenoma and benign Brenner tumor of the ovary [10,12].

## Competing interests

The author(s) declares that she has no competing interests.

## Authors' contributions

WAL performed the pathologic examination, researched the relevant literature and prepared the manuscript.

## Funding source

This research was conducted by the research funds of Dankook University in 2004.
